# A patient with hepatocellular carcinoma who developed invasive pulmonary aspergillosis after corticosteroid treatment

**DOI:** 10.1002/ccr3.4628

**Published:** 2021-08-10

**Authors:** Shintaro Sato, Sho Yamada, Tomotaka Nishizawa, Tomohiro Oba, Rie Kawabe, Hideaki Yamakawa, Keiichi Akasaka, Masako Amano, Hidekazu Matsushima

**Affiliations:** ^1^ Department of Respiratory Medicine Saitama Red Cross Hospital Saitama Japan

**Keywords:** cirrhosis, corticosteroid, hepatocellular carcinoma, invasive pulmonary aspergillosis

## Abstract

Lung or head and neck cancer have been indicated as solid cancers associated with invasive pulmonary aspergillosis (IPA), but the relationship with hepatocellular carcinoma (HCC) is unknown. We report a case of HCC in which the presence of cirrhosis and corticosteroid administration may have caused the development of IPA.

## Graphical abstract

The presence of hepatocellular carcinoma, cirrhosis of the liver, and corticosteroid administration predisposed the patient to the development of invasive pulmonary aspergillosis.

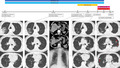

## 1 INTRODUCTION

Invasive pulmonary aspergillosis (IPA) has been traditionally described as the leading cause of invasive fungal disease in immunocompromised patients with hematologic malignancies associated with neutropenia and also those with solid organ transplantation, neoplasm, and human immunodeficiency virus (AIDS).^1^, ^2^ Recently, the profile of patients at high risk for IPA has expanded to include those with end‐stage chronic obstructive pulmonary disease (COPD) requiring corticosteroid therapy, severe cirrhosis, and those receiving immunosuppressive therapies (ie, monoclonal agents).^3^ However, to the best of our knowledge, there have been no reports of IPA associated with hepatocellular carcinoma. We report a case of recurrent hepatocellular carcinoma in which IPA developed after the start of corticosteroid therapy.

## 2 CASE HISTORY

**FIGURE 1:**
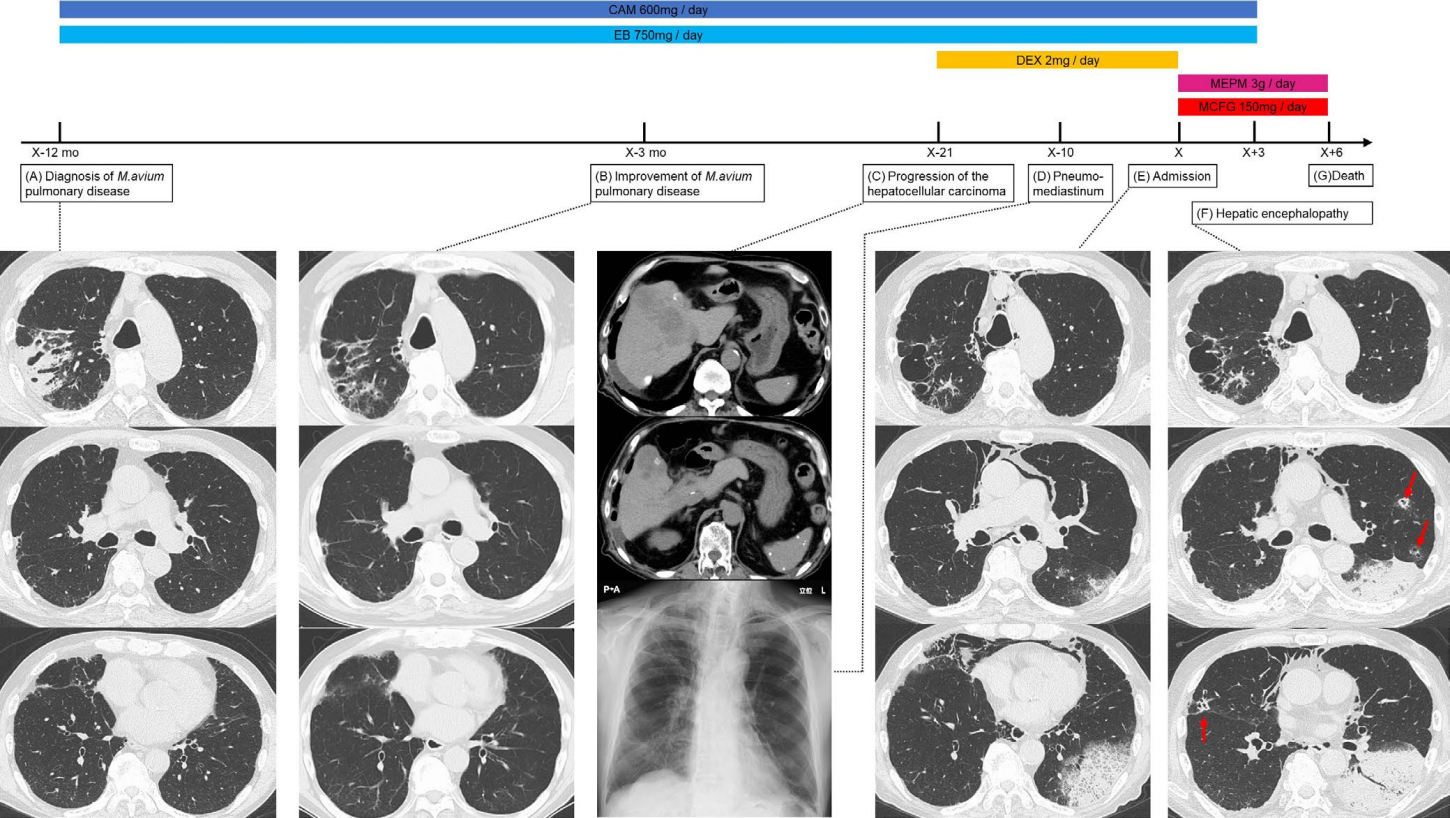
Clinical course. A, At the time of diagnosis of *Mycobacterium avium* pulmonary disease 12 months prior to admission, the patient had an infiltrative shadow in the right upper lobe with cavities and bronchiectasis. No obvious pulmonary lesions other than in the upper lobe were observed. B, Three months prior to admission, the infiltrative shadow had apparently resolved after 9 months of combination therapy of clarithromycin and ethambutol, but the cavities and bronchiectasis remained. C, Three weeks prior to admission, there was a growing hepatocellular carcinoma lesion in the right lobe of the liver with scattered areas of high density due to lipiodol infusion in the context of liver cirrhosis. D, Ten days before admission, chest X‐ray showed pneumomediastinum and subcutaneous emphysema. No pneumothorax or pulmonary lesions were found. E, On admission, the lung lesion of *Mycobacterium avium* in the right upper lobe remained unchanged, and extensive consolidation was observed from just below the pleura to the middle layer in the left lower lobe, and ground‐glass opacities were recognized at the margins. F, On the third day after admission, the infiltrative shadow had expanded in the left lower lobe and was accompanied by new cavitary nodules (arrows)

A 77‐year‐old Japanese man was referred to our hospital at X‐12 months for further examination of abnormal chest shadows. He had never smoked and had a 30‐year history of consuming 40 g/day of alcohol. He had been diagnosed as having hepatocellular carcinoma associated with alcoholic steatohepatitis at age 65 and underwent partial hepatectomy (S1/S6/S8), a total of four transcatheter arterial embolizations, and chemotherapy with sorafenib for postoperative recurrence. And he had only received the best supportive care for hepatocellular carcinoma from X‐12 months. Chest X‐ray and computed tomography (CT) showed an infiltrative shadow in the right upper lobe with cavities and bronchiectasis (Figure 1A), so we performed sputum examinations and identified *Mycobacterium avium* in different multi‐day specimens, thus resulting in a diagnosis of *M*. *avium* pulmonary disease. We started combination therapy of clarithromycin 600 mg/day and ethambutol 750 mg/day without rifampicin due to concerns about his hepatic functional reserve. Chest images showed apparent improvement in the infiltrative shadow at X‐3 months, but cavitary lesions and bronchiectasis remained in the same area (Figure 1B). At X‐21 days, he was prescribed 2 mg/day of dexamethasone by his palliative care physician to address his low appetite due to the progression of his hepatocellular carcinoma with cirrhosis (Figure 1C). At X‐10 days, he suddenly noticed swelling of his neck and visited the physician again, where a chest X‐ray revealed pneumomediastinum and subcutaneous emphysema with no apparent change in the lung field (Figure 1D). His dyspnea gradually worsened in the days that followed, and he was admitted to our hospital.

On physical examination, chest auscultation was normal and subcutaneous emphysema was palpable in the anterior neck. Chest CT revealed extensive infiltration from just below the pleura to the middle layer in the left lower lobe, and ground‐glass opacities were recognized at the margins (Figure 1E). Laboratory examination revealed significantly high serum levels of C‐reactive protein (10.2 mg/dL), β‐D‐glucan (804 pg/mL), and aspergillus antigen (>5.0) (Table 1). He also had alcoholic liver cirrhosis with a moderate Child‐Turcotte‐Pugh score and elevated α‐fetoprotein and protein induced by vitamin K absence, presumably due to the progressive hepatocellular carcinoma.

**TABLE 1:** Laboratory data at admission

Hematology				T‐Bil	2.4	U/L		Serology		
WBC	4330	/μL		AST	132	U/L		Aspergillus antigen	>5.0	
Neut	87.8	%		ALT	217	U/L		Aspergillus antibody	<4.0	
Lymp	9.2	%		LDH	615	U/L		β‐D‐glucan	804	pg/ml
Eo	0.0	%		ALP	269	U/L		AFP	1020	ng/ml
Baso	0.9	%		γGTP	194	U/L		PIVKA‐II	835	mAU/ml
RBC	478	/μL		BUN	47.6	mg/dL				
Hb	15.4	g/dL		Cr	0.92	mg/dL		Arterial blood gas analysis (room air)		
Plt	7.9	/μL		Na	134	mEq/L		pH	7.491	
				K	5.8	mEq/L		PaCO_2_	27.4	Torr
Biochemistry				Cl	101	mEq/L		PaO_2_	87.3	Torr
TP	5.6	g/dL		CRP	10.2	mg/dL		HCO_3_^−^	20.5	mmol/L
Alb	2.3	g/dL		NH_3_	150	μg/dL		BE	−1.2	mmol/L

Abbreviations: AFP, α‐fetoprotein; Alb, albumin; ALP, alkaline phosphatase; ALT, alanine aminotransferase; AST, aspartate aminotransferase; Ba, basophil; BUN, blood urea nitrogen; Cl, chloride; Cr, creatinine; CRP, C‐reactive protein; Eo, eosinophil; Hb, hemoglobin; K, serum potassium; LDH, lactate dehydrogenase; Ly, lymphocyte; Mo, monocyte; Na, serum sodium; Neut, neutrophil; NH_3_, ammonium hydroxide; PIVKA, protein induced by vitamin K absence; RBC, red blood cell; T‐Bil, total bilirubin; TP, total protein; WBC, white blood cell; γ‐GTP, γ ‐glutamyl transpeptidase.

We suspected bacterial or fungal pneumonia because of the acute onset and the lack of worsening of the right upper lobe lesion due to *M*. *avium* pulmonary disease, and we started treatment with meropenem 3 g/day and micafungin 150 mg/day. However, his respiratory and hepatic failure progressed rapidly with the expansion of the infiltrative shadow and new cavitary nodules (Figure 1F), and by the third day of hospitalization, he was unable to communicate due to hepatic encephalopathy and required the use of mask oxygen at 8 L/min. The rapid deterioration of his general condition made bronchoscopic evaluation difficult, and he died of multiple organ failure on the sixth day of hospitalization. The results of sputum tests submitted during hospitalization were received postmortem, and no bacteria, acid‐fast bacilli, or malignant cells were detected, but *Aspergillus terreus* was identified in cultures of several specimens, which ultimately led to the diagnosis of probable IPA as defined by the recent consensus definitions of invasive fungal disease from the European organization for research and treatment of cancer and the mycoses study group education and research consortium.^4^

## 3 DISCUSSION

We report a case of IPA occurring three weeks after the initiation of corticosteroid treatment for hepatocellular carcinoma. Most solid tumors associated with IPA are reported to be lung or head and neck cancers,^5^ but to the best of our knowledge, there have been no previous reports of cases of coexisting hepatocellular carcinoma. We suspect that the development of IPA was induced by the addition of corticosteroid to hepatocellular carcinoma in the background of cirrhosis, and prophylactic administration of antifungal drugs may be an option to consider when corticosteroid is used, especially in patients with cirrhosis.

IPA is recognized as an infection that occurs in patients with hematologic malignancies, especially during neutropenia, but in recent years, it has been estimated that 43%–80% of patients with IPA do not have hematologic malignancies.^6^ The non‐hematological populations at risk are diverse and include patients with solid organ transplantation, AIDS, solid tumors, COPD, and influenza infection. However, patients with these various conditions were frequently excluded from past studies of antifungal agents, and often, they do not meet the European Organization for Research and Treatment of Cancer/Invasive Infectious Diseases Study Mycoses Group diagnostic criteria of IPA, leaving several clinical aspects unexplored.^6^ Limited reports indicate that the prevalence of IPA in solid tumors ranges from 0.7 to 2.6%.^4^, ^5^ The most common type of cancer is lung cancer, followed by head and neck, breast, and gastrointestinal cancers, but there have been no reports of hepatocellular carcinoma. However, it was recently pointed out that cirrhosis is also one of the risk factors for the development of IPA,^7^, ^8^ and it is speculated that cirrhosis‐related immune dysfunction, which involves immunodeficiency and disturbance of specific immune system cells, including not only neutrophils and monocytes but also T and B cells, may be the root cause.^9^ Therefore, we believe that evaluating the complication of cirrhosis in patients with hepatocellular carcinoma is important to accurately assess the risk of IPA.

Corticosteroid treatment has been widely reported to be a risk factor for the development of IPA, with a higher incidence in non‐neutropenic patients, critically ill non‐cancer patients, and patients with COPD, cirrhosis, and AIDS.^4^, ^10^, ^11^, ^12^, ^13^ The suppression of neutrophil function by corticosteroid is a contributing factor in the promotion of *Aspergillus* colonization and infection.^14^ It is also noted that approximately two‐thirds of patients who develop IPA while on corticosteroid do not have a fever, which can easily lead to a delay in diagnosis.^15^ Corticosteroid can place patients at high risk of developing IPA at cumulative doses of more than 700 mg of prednisolone equivalent,^15^ and careful consideration should be given to long‐term or high‐dose corticosteroid treatment in general. The use of prophylactic antifungal drugs should be considered as an option if the risk of developing IPA is considered high,^6^ and risk assessment must take into account both the underlying disease and the treatment received. Our patient developed IPA shortly after the start of corticosteroid therapy, and we hypothesize that the presence of both cirrhosis and corticosteroid use may have triggered the development of IPA. However, it is interesting to note that the patient developed pneumomediastinum and subcutaneous emphysema shortly before the onset of IPA. A definitive interpretation of this process is difficult to make, but possible causes include tissue fragility due to corticosteroid and bronchial wall damage due to a central airway lesion called pseudomembranous tracheobronchitis caused by *Aspergillus*.^16^, ^17^ Bronchoscopy could have been useful in evaluating airway lesions, although it could not be performed in this case due to the extremely poor general condition.

Although the association of nontuberculous mycobacterium (NTM) infection with pulmonary aspergillosis, especially chronic progressive pulmonary aspergillosis, has been noted before,^18^, ^19^, ^20^ few reports have pointed out an association between NTM and IPA. The CT scans of our patient showed predominantly infiltrative shadows and ground‐glass opacities without centrilobular nodules, and we presumed these findings to indicate a vascular‐invasive IPA rather than an airway‐invasive IPA. The chest images also showed that the right upper lobe lesion caused by the *M*. *avium* pulmonary disease remained improved whereas new left lower lobe infiltrative shadows appeared in a normal lung with no pre‐existing lesions, suggesting that IPA may have occurred without apparent association with the *M*. *avium* pulmonary disease. We believe that *M*. *avium* pulmonary disease is unlikely to have played a significant role in the development of IPA in this case and speculate that the effects of cirrhosis and corticosteroid, which can affect systemic immune function, may have been more significant than the localized lung disease.

We often use corticosteroid to reduce symptoms in the palliative care of patients with solid tumors, and in many of these attempts, the corticosteroid can be effective.^21^, ^22^, ^23^ However, the overall picture of adverse events from corticosteroid is not fully clarified, and it is also noted that the implementation of antifungal prophylaxis is low in solid tumors compared to hematologic malignancies.^24^, ^25^ The prognosis of IPA is extremely poor, and clinicians need to discuss with patients whether to provide prophylaxis when using corticosteroid or to conduct surveillance for the early detection of IPA.

In conclusion, we experienced a case of invasive pulmonary aspergillosis in a patient with hepatocellular carcinoma. The patient had underlying alcoholic liver cirrhosis, and the initiation of systemic corticosteroid may have triggered the development of IPA. When starting corticosteroid in patients with hepatocellular carcinoma, it is important to confirm the presence of cirrhosis and, if necessary, to provide prophylactic treatment for IPA with antifungal agents and to monitor the incidence of IPA.

## CONFLICT OF INTEREST

The authors have no conflict of interests to disclose.

## AUTHOR CONTRIBUTIONS

SS and SY involved in the diagnosis and treatment of the case, and prepared and drafted the manuscript. TN, TO, RK, HY, KA, MA, and HM assisted in the preparation of the manuscript. All authors reviewed and approved the final manuscript.

## ETHICAL APPROVAL

All procedures were under the ethical standards of the local ethics committee.

## Data Availability

The authors declare that all data supporting the findings of this study are available within the article.
